# Supplementary material for: The causal relationship between the human gut microbiota and pyogenic arthritis: a Mendelian randomization study
Source: Front Cell Infect Microbiol. 2024 Nov 26;14:1452480. doi: 10.3389/fcimb.2024.1452480 (PMC11629706; doi:10.3389/fcimb.2024.1452480)
Supplement: Supplementary file 1 [file DataSheet1.zip › mendelian test/ebi-a-GCST90027539.csv_leave_one_out.pdf]

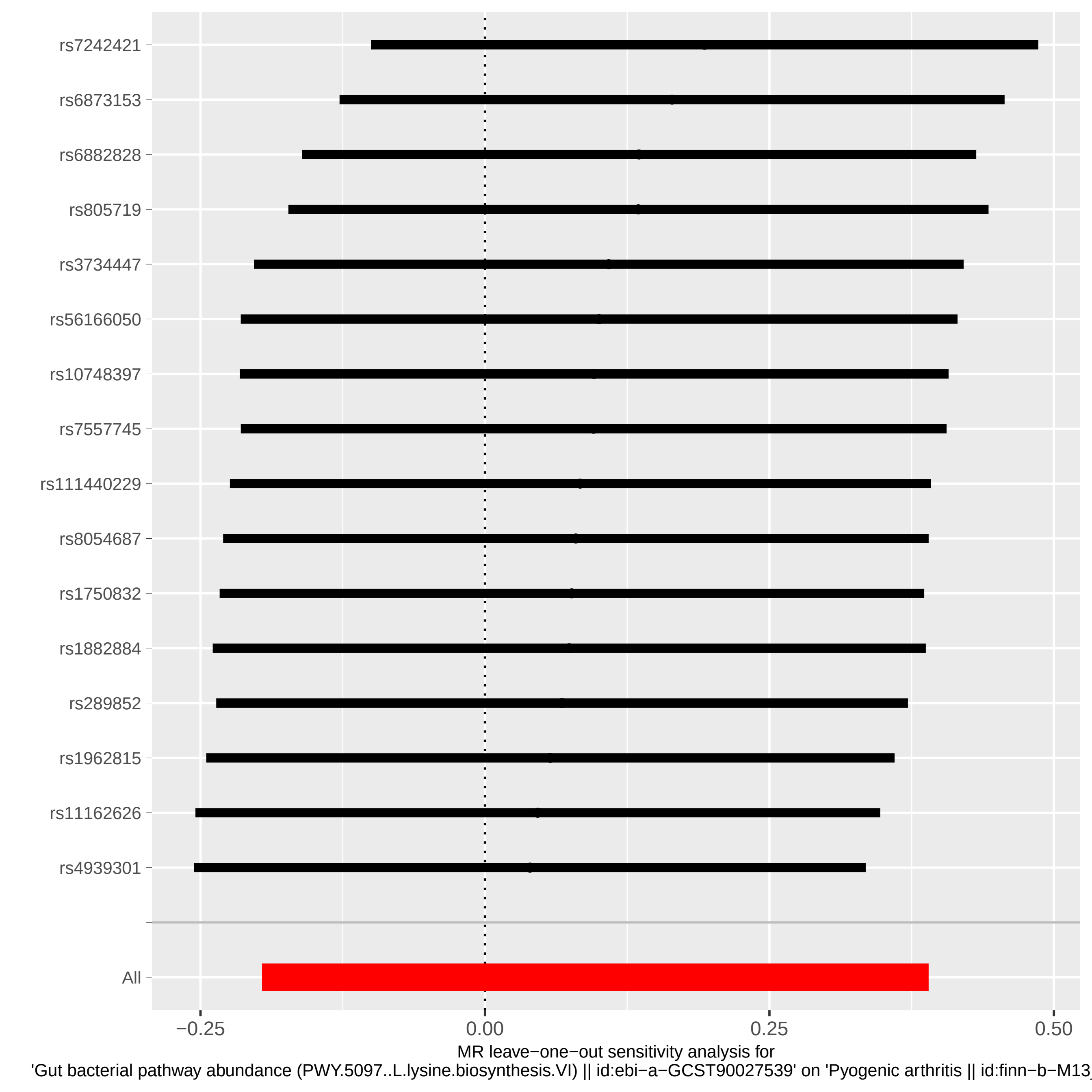
rs7242421
rs6873153
rs6882828
rs805719
rs3734447
rs56166050
rs10748397
rs7557745
rs111440229
rs8054687
rs1750832
rs1882884
rs289852
rs1962815
rs11162626
rs4939301
All
−0.25
0.00
0.25
0.50
MR leave−one−out sensitivity analysis for
'Gut bacterial pathway abundance (PWY.5097..L.lysine.biosynthesis.VI) || id:ebi−a−GCST90027539' on 'Pyogenic arthritis || id:finn−b−M13_
